# Tumor resistance to vascular disrupting agents: mechanisms, imaging, and solutions

**DOI:** 10.18632/oncotarget.6999

**Published:** 2016-01-22

**Authors:** Wenjie Liang, Yicheng Ni, Feng Chen

**Affiliations:** ^1^ Department of Radiology, The First Affiliated Hospital, School of Medicine, Zhejiang University, Hangzhou, Zhejiang, China; ^2^ Radiology Section, University Hospitals, University of Leuven, Leuven, Belgium

**Keywords:** vascular disrupting agents, resistance, imaging, solutions

## Abstract

The emergence of vascular disrupting agents (VDAs) is a significant advance in the treatment of solid tumors. VDAs induce rapid and selective shutdown of tumor blood flow resulting in massive necrosis. However, a viable marginal tumor rim always remains after VDA treatment and is a major cause of recurrence. In this review, we discuss the mechanisms involved in the resistance of solid tumors to VDAs. Hypoxia, tumor-associated macrophages, and bone marrow-derived circulating endothelial progenitor cells all may contribute to resistance. Resistance can be monitored using magnetic resonance imaging markers. The various solutions proposed to manage tumor resistance to VDAs emphasize combining these agents with other approaches including antiangiogenic agents, chemotherapy, radiotherapy, radioimmunotherapy, and sequential dual-targeting internal radiotherapy.

## INTRODUCTION

The emergence of small molecule agents that target the tumor vasculature is an advance in the treatment of malignant solid tumors [1-3]. Antivascular drugs can be divided into two types according to the molecular target strategy: antiangiogenic agents and vascular disrupting agents (VDAs). Antiangiogenic agents act on the signaling pathways between tumor cells, endothelial cells (ECs), and stromal cells to inhibit the formation of new blood vessels [4, 5]. The United States Food and Drug Administration (FDA) has approved several antiangiogenic agents such as bevacizumab, sunitinib, and sorafenib. However, they are not the focus of this review. Small molecule VDAs kill tumor cells by inducing rapid and selective shutdown of tumor blood flow. Although VDAs have not been approved by the FDA, they have shown significant therapeutic potential and are a focus of current research [6]. VDAs mainly include tubulin-binding agents such as combretastatins and drugs related to 5, 6-dimethylxanthenone-4-acetic acid (DMXAA). Combretastatin A-4 3-O-phosphate (CA4P; the lead combretastatin) and its derivatives such as the more effective CA1P (Oxi4503) and synthetic analogue AVE8062 are currently in preclinical and clinical development [6]. Despite the encouraging therapeutic effects of VDAs, clinical trials have shown that residual marginal tumor cells are less sensitive to CA4P [7] and can survive nutrient-deficient conditions. Moreover, the viable marginal tumor cells are a major cause of recurrence and metastasis [6, 7] and are indicative of resistance to VDAs [8, 9].

In this review, we summarize the mechanisms involved in the resistance of solid tumors to VDAs, the role of imaging markers in visualizing and understanding resistance, and the various solutions proposed to overcome tumor resistance to VDAs.

## MECHANISMS OF SOLID TUMOR RESISTANCE TO VASCULAR DISRUPTING AGENTS

### Residual viable tumor rim and hypoxia

The VDA-induced rapid shutdown of the tumor vasculature leads to massive central tumor necrosis. The entire tumor, including the necrotic portion and residual viable rim, become hypoxic due to the reduced blood supply [10, 11]. Thus, hypoxia may be one of the key factors that contribute to VDA resistance. Since the residual viable tumor cells may obtain oxygen and nutrients from nearby blood vessels in the normal tissue, they can metabolically adapt to hypoxic conditions and become hypoxia-tolerant. According to El-Emir et al. [11], hypoxia reaches a maximum at both the central and peripheral portions of a tumor 1 hour after CA-4P administration. It is then alleviated after 24 hours and is restricted to regions adjacent to the central necrotic area. Hypoxia results in upregulation of the expression of hypoxia inducible factor 1α (HIF-α), which activates transcription of a large panel of genes involved in angiogenesis and increases the levels of circulating proangiogenic cytokines including vascular endothelial growth factor (VEGF) and stromal derived factor 1α (SDF-1α) [12]. Experiments with tumor-bearing animals have confirmed that after VDA treatment, the levels of VEGF and basic fibroblast growth factor increased significantly [13-15]. Newly formed tumor vessels provide nutrients for residual peripheral tumor cells and promote growth and proliferation. Under hypoxic conditions, HIF-1α is also involved in glycolysis and the microenvironment acidification of tumors, which influence both cell survival and cell death. Thus, HIF-1α drives tumorigenesis and metastasis [16, 17].

Long-term follow-up of clear-cell carcinoma patients who underwent radical nephrectomy showed that VEGF and HIF-1α were closely related to prognosis, and that VEGF was an independent predictor of prognosis [18]. The enhanced invasiveness of HIF-1α-induced tumor cells has been demonstrated in vitro [19]. Another study confirmed that HIF-1α could independently increase the malignant potential of a hypoxia-tolerant tumor cell line [20]. Under anoxic conditions, tumor cells have been shown to express C-X-C chemokine receptor type 4, which may also enhance the malignant potential of tumor cells via the relevant signaling pathways [21-23].

### Tumor-associated macrophages

Tumor-associated macrophages (TAMs) are circulating monocyte- or resident tissue macrophage-derived cells. Although TAMs are thought to promote angiogenesis, they may function antagonistically (i.e., exert either pro- or antitumor effects). For instance, Welford et al. [24] showed that there was an increase in SDF-1- and TIE2-expressing macrophages (TEMs), a proangiogenic subset of TAMs, with CA4P treatment, suggesting that TEMs could limit the therapeutic efficacy of CA4P in tumor-bearing mice. Other studies have demonstrated similar results [25, 26]. However, Jassar et al. [27] showed that DMXAA directly activates TAMs and induces an effective antitumor response in murine models of lung cancer and mesothelioma. Wallace et al. [28] confirmed the finding that DMXAA activates dendritic cells and induces cytotoxic antitumor effects. Therefore, TAMs may have different roles with respect to the therapeutic effects of VDAs.

### Bone marrow-derived circulating endothelial progenitor cells

Circulating endothelial progenitor cells (CEPs) are bone marrow (BM)-derived immature endothelial cells in the peripheral blood and account for a small proportion of circulating endothelial cells (CECs). CEPs are defined by cell surface expression of vascular endothelial cadherin, dim CD31 and CD45, CD34, CD133, and vascular endothelial growth factor receptor-2 (VEGFR-2 or KDR in humans; flk-1 in mice) [29-31]. The VDA-induced mobilization of BM-derived CEPs was first reported in an animal study [32]. After tumor-bearing mice were treated with the VDA derivative OXi-4503, the CEP count increased significantly and reached a peak 4 hours after treatment. The CEPs appeared to home to sites of viable tumor cells where they incorporated into the endothelial cells of tumor vessels and promoted tumor vasculogenesis [32]. Thus, tumors may become resistant to VDAs through a CEP-related mechanism.

The phenomenon of VDA-induced CEP mobilization has also been reported in humans. For example, Farace et al. [33] determined that CEP levels increased to various extents 3 to 7 days after treatment with AVE8062 in patients with solid tumors. In contrast to the findings of Shaked et al. [29] and Farace et al. [32, 33], Taylor et al. [8] observed two spikes in CEP levels in different tumor-bearing animal models in which the animals were treated with VDAs. An early increase in CEP levels was observed 2 to 4 hours after CA4P treatment, and a rapid and significant rise in CEP levels occurred 72 or 96 hours after CA4P treatment compared to the levels after 4 hours. The early CEP peak suggested a general host response to CA4P because it was detected in both tumor- and non-tumor-bearing animals. However, the late increase in CEPs reflected the specific VDA-induced tumor responses of vascular repair and regrowth because the delayed increase in CEP levels was exclusively observed in tumor-bearing animals, and was verified in different animal species and tumor models [8]. The second spike in CEPs reportedly parallels elevated levels of serum granulocyte colony-stimulating factor (G-CSF), matrix metallopeptidase-9 (MMP-9), and bone marrow stromal-derived factor-1 (SDF-1) [34-37], suggesting that these three proteins could promote the delayed mobilization of CEPs to the peripheral circulation after VDA treatment.

The theory of VDA-induced CEP mobilization has been challenged by the conflicting results of other studies [8, 38, 39]. Ziegelhoeffer et al. [40] investigated the relationship between BM-derived cells expressing enhanced green fluorescent protein (GFP) and new vessels in murine hind limb ischemia and tumor models, and failed to find any endothelial or smooth muscle cells displaying GFP signals. Although some GFP-positive cells were observed in the local ischemic area, they were identified as fibroblasts, pericytes, or leukocytes rather than endothelial cells. They concluded that BM-derived cells do not incorporate into the adult growing vasculature, but may function as supporting cells. Göthert et al. [41] traced the origin of the tumor endothelium in a transgenic murine model and determined that no BM-derived cells contributed to the tumor endothelium.

Purhonen et al. [39] induced angiogenesis in four different genetically tagged mouse models and studied the mobilization of BM-derived cells to the endothelium activated by VEGF and tumors. Interestingly, they observed many BM-derived cells surrounding endothelial cells in blood vessel walls, but did not observe incorporation of these cells into the endothelium. They also did not observe mobilization of BM-derived VEGFR-2^+^ cells into the circulation and concluded that tumor growth does not require BM-derived CEPs. Many of these studies challenged the theory that CEPs are involved in tumor vasculogenesis, although VDAs were not used to induce BM-derived CEP mobilization in these studies. However, in a rat liver xenograft tumor model treated with ZD6126, Chen et al. [12] found that VDA treatment did not induce a significant increase in CEPs or plasma SDF-1α 4 hours or 2 days following therapy.

**Figure 1:**
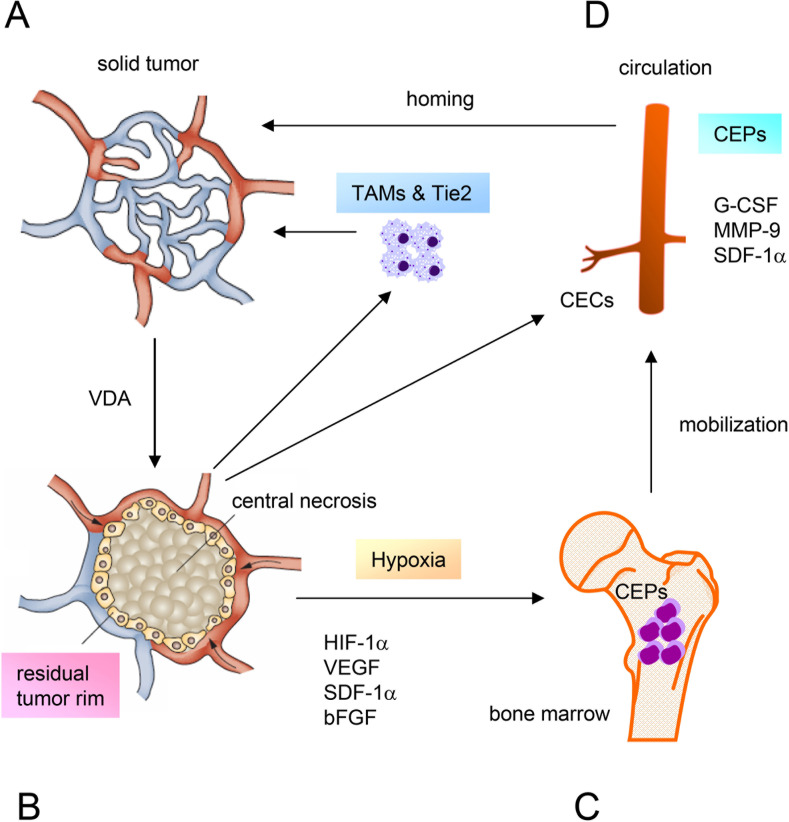
Diagram illustrating the mechanisms of tumor resistance to vascular disrupting agents **A.** Solid tumor treated with a VDA. **B.** VDA-induced central necrosis and a residual viable tumor rim. Hypoxia upregulates the expression of HIF-1α, which increases the levels of a number of circulating proangiogenic cytokines and chemokines. **C.** Activation of proangiogenic pathways mobilizes BM-derived CEPs into the circulation, and is accompanied by increases in serum G-CSF, MMP-9, and SDF-1α. CEC levels increase due to irreversible injury of the tumor vasculature caused by VDA treatment. **D.** CEPs are attracted to the tumor where they incorporate into the endothelial cells of tumor vessels and promote vasculogenesis. The increased levels of TAMs and TEMs induced by VDAs limit the therapeutic efficacy. Thus, all of these factors contribute to the resistance of tumors to VDAs. Adapted from Health VL, Bicknell, R. Anticancer strategies involving the vasculature. Nature Reviews Clinical Oncology. 2009; 6: 395-404 (Figure 1) and Schmid MC, Varner JA. Myeloid cells in the tumor microenvironment: modulation of tumor angiogenesis and tumor inflammation. Journal of Oncology. 2010; 201026: 1-10 (Figure 2).

There are a number of possible explanations for the above-mentioned conflicting results. First, there may have been some false-positives regarding the identification of CEPs in earlier studies due to the signal superimposition of vessel wall CEPs and adjacent hematopoietic cells [40]. Second, as reported in some studies [39, 40], BM-derived cells were recruited only as perivascular supporting cells or pericytes. They occasionally expressed CEP markers, but did not form part of the endothelium during angiogenesis. Therefore, pericytes that originated from hematopoietic cells may have been misidentified as BM-derived CEPs. Third, several reports have indicated that mobilization of BM-derived CEPs only occurs in certain tumors [34], and little is known about which types of tumors are mainly reliant on CEPs for growth [31]. In addition, the VDA-induced mobilization of CEPs has been observed in murine models and human studies [32, 33], but not in rat studies [12]. Therefore, it is still unclear whether the response of CEPs to VDA treatment is species-dependent.

**Figure 2:**
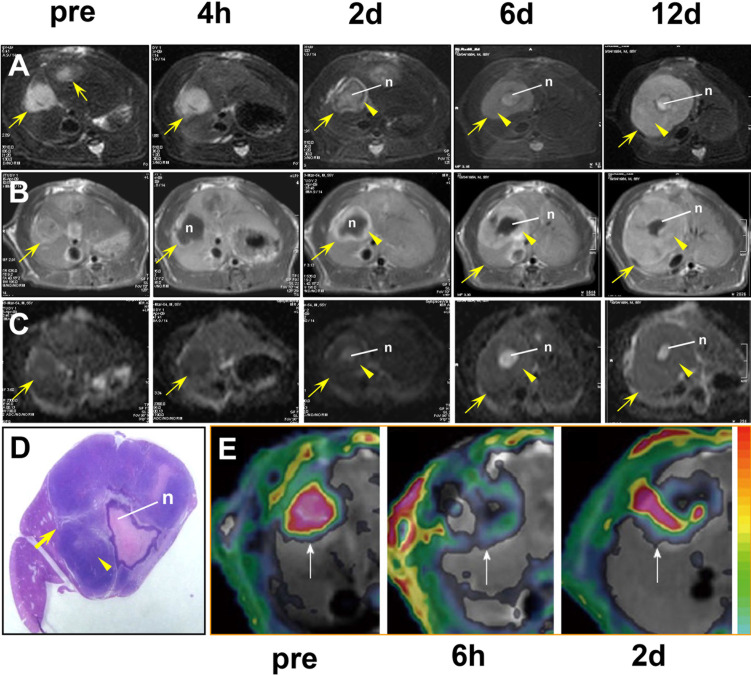
Imaging of tumor resistance to vascular disrupting agents in a rat liver tumor model **A.** MRI T2WIs show the tumor (arrow), central necrosis (n), and the viable rim (arrowhead) 2 days after ZD6126 treatment. Note the rapid regrowth of the tumor rim from 4 hours (h) to 12 days (d). **B.** On CE-T1WIs, the tumor rim (arrowhead) exhibits hyperintense enhancement after injection of a contrast agent, indicating it is rich in blood vessels. **C.** On ADC maps derived from diffusion-weighted MR images, the tumor rim (arrowhead) exhibits a decreased ADC, indicating increased cellularity due to tumor regrowth, with an elevated ADC in the central necrotic area (n). **D.** MRI findings of tumor rim enlargement (arrowhead) and central necrosis (n) 12 days after treatment are confirmed in the macroscopic tumor section. **E.** Dynamic changes in K^trans^ in a liver tumor model in another rat. The tumor (arrows) exhibits an abundant blood supply with high K^trans^ before treatment (pre). Six hours after CA4P treatment, vascular shutdown is indicated by a low K^trans^ in the central region surrounded by residual tumor at the periphery with a moderate K^trans^. Two days after treatment, tumor recurrence is evident at the periphery with a rebounding K^trans^. Figure 2E was reprinted and adapted with permission from Wang HJ, Marchal G, Ni Y. Multiparametric MRI biomarkers for measuring vascular disrupting effect on cancer. World J Radiol. 2011; 3: 1-16.

Mature CECs, which are usually defined phenotypically by the expression of membrane glycoprotein CD146, are a newly recognized population of non-hematopoietic cells in the blood [42]. As CEPs, they are rare in the blood of healthy individuals (0.01%), and are derived from existing vasculature. They may be sloughed off in a wide variety of pathological conditions including inflammatory, infectious, and vascular diseases. Therefore, an increase in CECs has been reported as a biomarker for assessing vascular insult. Beaudry et al. [43] reported that mature CECs were increased in mice with Lewis lung carcinoma 3 days after treatment with a VDA (ZD6126). Beerepoot et al. [44] also observed a VDA-induced increase in CECs in a clinical study. In another study of patients with advanced solid tumors [45], 18 out of 19 (95%) patients showed a significant increase in CECs 2 to 8 hours after infusion of ZD6126. A rapid increase in the CEC level could also be induced by treatment with taxane-based chemotherapy in patients with various solid tumors. Since no changes in CEC levels were detected in non-tumor-bearing VDA-treated mice or tumor-bearing vehicle-treated mice [8, 43], it was suggested that the increase in CEC level was the direct consequence of irreversible VDA-induced injury to the tumor vasculature [46]. Based on these findings, Bhatt et al. [47] hypothesized that the early increase in CECs induced by VDA therapy represents a parallel increase in the number of apoptotic CECs. Despite these findings, it is not clear whether CECs may be mobilized from the bone marrow by cytokines [45] and enhance tumor angiogenesis [48].

## IMAGING BIOMARKERS OF TUMOR RESISTANCE TO VASCULAR DISRUPTING AGENTS

Advances in the development of new anti-vascular therapies for solid tumors highlight the need for non-invasive imaging methods to evaluate therapeutic efficacy. Magnetic resonance imaging (MRI) is an established tool for the in vivo monitoring of anatomical and functional changes in tumors after treatment with novel molecular targeting drugs. MRI has superb soft tissue contrast, excellent temporal and spatial resolution, and is noninvasive. A multi-parametric MRI approach has been widely used to evaluate the therapeutic effects of VDAs, and typically includes the following imaging sequences: conventional anatomical imaging (i.e., T1-weighted imaging [T1WI] and T2-weighted imaging [T2WI]), functional imaging such as diffusion-weighted imaging (DWI), T1-weighted dynamic contrast-enhanced MRI (DCE-MRI), T2*-weighted dynamic susceptibility contrast-enhanced MRI (DSC-MRI), and contrast-enhanced T1WI (CE-T1WI). For a basic understanding of the MRI and sequences, please refer to detailed explanations elsewhere [49, 50].

A variety of imaging markers can be used to evaluate tumor resistance to VDAs. Although a single-dose of a VDA can cause extensive central necrosis of the tumor, a small solid rim always remains in the periphery, even in the most responsive tumors [6]. In preclinical studies, the viable tumor rim appeared 2 to 3 days after CA4P or ZD6126 treatment. The initial rim is typically several millimeters in width and will grow in a centrifugal manner over time toward the necrotic area, while the tumor volume may remain unchanged. The residual rim has been histologically verified to be the regrowth of tumor cells. On MRI, an enhanced rim on CE-T1WI after intravenous injection of a contrast agent reflects the sparing of viable tumor cells.

In addition to this morphological marker, functional imaging markers can also be used to evaluate tumor resistance to VDAs. DWI is a MRI technique that can be used to quantify the mobility of water molecules in vivo. By measuring the DWI-derived apparent diffusion coefficient (ADC) of the tissue, quantitative information on the movement of water molecules can be obtained. Regrowth of tumors that are resistant to VDAs may increase the cellularity, and the interstitial pressure can also be high [51]. This results in the restricted diffusivity of water molecules and a lower ADC in tumors compared to previous values or the pretreatment ADC. Thus, a decrease in the ADC of the tumor after VDA treatment is indicative of regrowth [52].

Tumor blood vessels are immature and disorganized, often with poorly developed endothelial cell-lined basement membranes. Therefore, they have high permeability and high interstitial fluid pressure. This intrinsic feature of the tumor vascular can be evaluated using a parameter of permeability (i.e., K^trans^ [unit/min], the volume transfer constant of the contrast agent derived from DCE-MRI). An increase in K^trans^ can be an indicator of VDA-resistance. Increased interstitial fluid pressure can be assessed using another parameter, Ve (unit %), the extravascular extracellular volume fraction. Additionally, blood flow in the tumor vascular network is heterogeneous, low, and can be intermittent, although the blood volume may increase with tumor regrowth.

As an example, despite the rapid disruption of tumor blood vessels and extensive necrosis after treatment with VDAs, a ring of viable tumor cells has invariably been shown to appear at the tumor periphery after 2 to 3 days in a variety of preclinical tumor models [6, 51, 53-56]. The rim becomes more significant 9 to 12 days after treatment [12, 51] and it can remain even after a third administration of CA4P [53]. During this process, the ADC and blood perfusion-related parameters (K^trans^, ADC_perf_, initial slope) show a transient reduction from 1 to 6 hours as a result of the shutdown of blood vessels. This is followed by an increase in the ADC primarily in the center of the tumors at approximately 2 days, which is indicative of necrosis [51]. However, the decrease in the ADC and increase in K^trans^, blood flow, and blood volume at the tumor periphery from 2 to 9 or 12 days suggests the early recovery of perfusion and relapse of the tumor [12, 51]. All of these imaging markers of tumor resistance to VDAs have been found to be consistent with tumor relapse in the periphery and confirmed by histopathological examination.

Since genotypic heterogeneity within a solid tumor can greatly influence tumor growth and therapeutic response [57], spatial heterogeneity is common between and within tumors. This prevents adequate evaluation of therapeutic effects and is a key point in tumor resistance [58, 59]. Conventional imaging measurements using only the tumor size or average parameter values neglect the rich spatial information inherent to tumors. Additional quantitative MRI approaches have been used to overcome this problem. For instance, histogram analysis can be used to characterize and compare the distribution of tumor imaging markers by quantifying the number of pixels according to each intensity level in the entire tumor. Baek et al. [60] reported that the percent change of histogram-derived skewness and kurtosis of perfusion can be used to differentiate early tumor progression from pseudoprogression in patients with glioblastoma. In a study by Ahn et al. [61], histogram analysis of ADC maps was successfully used to differentiate histological grades of head and neck squamous cell carcinoma. The functional diffusion map (fDM) derived from DWI [62, 63], or parameter response maps (PRMs) derived from DSC-MRI [64] or DCE-MRI [65], are voxel-based analytical methods that use registered functional parameter maps before and after treatment. They allow categorization of the individual tumor voxel into three groups (increased, unchanged, decreased) based on the extent of the changes in parameter values during therapy. In an animal tumor model, fDM has demonstrated the potential to detect the emergence of resistance in real-time [66]. Thus, spatially heterogeneous regions that exhibit either responsive or early emergence of VDA-resistant cell populations within a tumor can be quantified by fDM or PRM.

**Figure 3:**
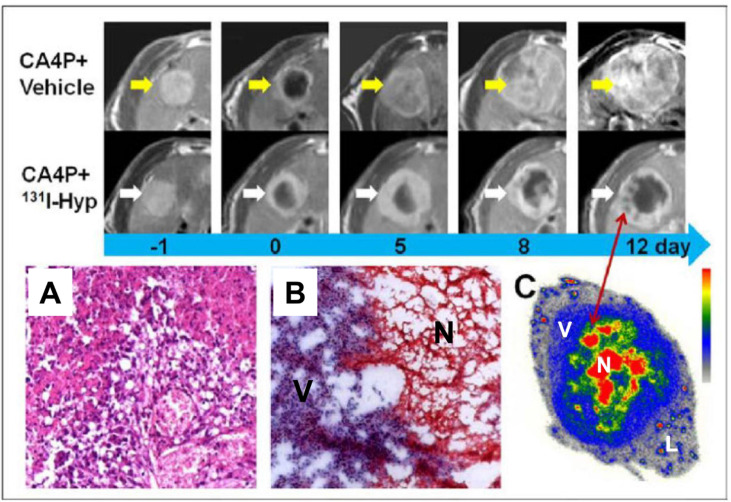
CA4P in combination with necrosis-targeted radiotherapy with ^131^I-Hyp-labeled hypericin First two rows. MR images of representative tumors from the two groups show increased tumor volume as well as intratumoral necrosis. On day 0, a hyperintense rim is observed surrounding the hypointense necrotic tumor. As compared with the CA4P control group, tumor growth in the ^131^I-Hyp group was much slower, with intratumoral necrosis present until 12 days. **A.**, **B.** Photomicrographs of 5 μm tumor sections sampled from the interface between necrotic (N) and viable tumor cells (V). In the CA4P control group (A), viable and dead cells coexist with new vessels. Ionizing radiation of tumor cells exposed to ^131^I-Hyp causes marked cell death characterized by cell membrane damage and extensive tumor damage (B). **C.** Twelve days after administration of ^131^I-Hyp, foci indicative of radiation-induced cell death are observed inside the viable tumor rim in MR images (red arrow), which corresponded to nests of relative high radioactivity in autoradiograms of the same sections. The liver (L) displays much lower radioactivity compared to the viable tumor (V), and the intratumoral necrosis (N). Reprinted with permission from Li J, Cona MM, Chen F, Feng Y, Zhou L, Yu J, Nuyts J, de Witte P, Zhang J, Himmelreich U, Verbruggen A, Ni Y. Theranostics. 2012; 2: 1010-1019.

**Table 1:** Representative solutions for tumor resistance to vascular disrupting agents (VDAs)

Author	Year	Subject	VDA	Combination therapy	Sequence of therapies	Outcomes	Ref
Siemann DW et al	2004	nude mice	ZD6126	Antiangiogenic agents (AA): ZD6474	AA + VDA	tumor growth delay	[65]
Shaked Y et al	2006	nude mice	Oxi-4503	AA: DC101	AA + VDA	reduction in tumor rim and blood flow	[29]
Chen F et al	2012	rat	ZD6126	AA: Thalidomide	AA + VDA	reduction in tumor rim and hemodynamic index	[10]
Siemann DW et al	2002	nude mice	DMXAA, CA4P	Chemotherapy (Chem): cisplatin, cyclophosphamide	Chem + VDA	extensive hemorrhagic necrosis, dose dependent tumor cell death	[88]
Martinelli M, et al	2007	nude mice	ZD6126	Chemo: paclitaxel	VDA + Chem	50-57% tumors regressing	[92]
Daenen LG et al	2009	nude & SCID mice	OXi4503	Chemo: Low-dose metronomic cyclophosphamide	Chem + VDA	decrease of tumor rim and marked suppression of tumor growth	[71]
Li J et al	1998	mice	CA4P	Radiotherapy (Radio)	Radio + VDA	enhancements in tumor cell killing & antitumor effects of radiotherapy	[100]
Murata R et al	2001	mice	DMXAA	Radio	Radio + VDA	enhancement of tumor radiation damage	[91]
Ng QS et al	2012	phase Ib trial in NSCLC patients	CA4P	Radio	Radio + VDA	well tolerated in most patients; 7 responses out of 18 patients	[72]
Iversen AB et al	2013	mice	DMXAA CA4P OXi4503	Radio: single or fractionated radiation	Radio + VDA VDA + Radio	increased antitumor effects; increased response only seen in OXi4503	[101]
Pedley RB et al	2001	nude mice	CA4P	Radioimmuno- Therapy (RIT): ^131^I labled-antibody-targeted	RIT + VDA	complete tumor cures in five of six mice	[74]
Meyer T et al	2009	phase I trial in advanced cancers	CA4P	RIT: ^131^I labled- antibody-targeted	RIT + VDA	a partial response shown in one out of ten patients	[73]
Li J et al	2011	rat	CA4P	^131^I labled necrosis targeting hypericin (^131^I–hypericin)	VDA + ^131^I-hypericin	kill of residual tumor cells & inhibited tumor regrowth	[78]
Shao H et al	2015	rabbit	CA4P	^131^I–hypericin	VDA + ^131^I-hypericin	well inhibited viable tumor rims & prolonged tumor doubling time	[80]

## SOLUTIONS FOR TUMOR RESISTANCE TO VASCULAR DISRUPTING AGENTS

More potent VDAs have been developed to increase the therapeutic efficacy. For example, CA1P, a second-generation small molecule derivative of CA4P, has been shown to induce a smaller viable tumor rim. Another synthetic CA4P derivative, AVE8062, has also demonstrated enhanced antitumor activity by more substantially decreasing tumor blood flow [6, 67, 68]. Many strategies have been proposed to improve VDA effectiveness. Current efforts have predominantly involved combining VDAs with other approaches such as antiangiogenic agents [12, 32, 68-71], conventional chemotherapy [72-75], radiotherapy [67, 76], radioimmunotherapy [77-79], or dual-targeting therapy [80-84].

### Vascular disrupting agents combined with antiangiogenic agents

In theory, VDAs and antiangiogenic agents should act synergistically because VDAs induce acute vascular shutdown and antiangiogenic agents inhibit the growth of new tumor vessels. Many studies have confirmed this hypothesis. Siemann et al. [69] treated human renal cell carcinoma and Kaposi's sarcoma in mice with a combination of ZD6126 (vascular disrupting) and ZD6474 (antiangiogenic). The combined therapy resulted in a remarkable delay in tumor growth compared to either agent alone. Additionally, Shaked et al. [32] treated tumor-bearing mice with DC101, an antiangiogenic agent, followed by OXi-4503, a VDA, and observed a significant reduction in tumor rim size and blood flow. This was a result of the suppression of CEP levels and mobilization by DC101 [85], which would otherwise home to the viable tumor rim and contribute to angiogenesis. In a rodent liver tumor model, Chen et al. [12] studied combined treatment with ZD6126 and thalidomide, an antiangiogenic agent [86]. They observed cumulative tumor apoptosis or necrosis, and consequently a decrease in the viable tumor rim. The combined approach also prolonged the duration of the reduction in K^trans^ in the tumor, and improved the hemodynamic index. This was most likely a result of the transient normalization of tumor vessels [87] induced by thalidomide. However, Chen et al. did not observe acute mobilization of CEPs after ZD6126 treatment [10].

An increase in CECs can result from a direct vascular insult, and a VDA-induced rise in CECs has been observed in both an animal study [43] and in a clinical trial [44]. However, the combined use of an antiangiogenic agent with a VDA did not suppress CEC levels. Instead, CEC levels could increase [47], although the rapid rise in CEPs could be blocked [32]. Thus, both CECs and CEPs may be used to monitor the therapeutic effects of anti-vascular treatments.

### Vascular disrupting agents combined with chemotherapy

Based on experimental observations, VDAs and chemotherapeutic agents can be categorized as microtubule-binding agents, and differences in therapeutic effects mainly depend on their tubulin binding sites and the duration of binding. For instance, most VDAs reversibly bind to the colchicine site of tubulin, whereas vinblastine and paclitaxel bind to the vinca alkaloid and taxane sites, respectively, and result in persistent inhibition of mitotic processes and proliferation [67].

Several mechanisms have been proposed to justify the combined regimen of a VDA and chemotherapy. First, because VDAs and cytotoxic agents target different components of tumors (i.e., the tumor vasculature and proliferating tumor cells, respectively), complementary benefits are possible [73]. In a Calu-6 model, ZD6126 combined with cisplatin resulted in enhanced delay of tumor growth [72]. A CA4 derivative, AVE8062, in combination with docetaxel significantly inhibited the growth of chemotherapy-resistant ovarian cancers and prolonged survival in HeyA8-injected mice [88]. The combination of CA4P with liposomal doxorubicin resulted in the greatest delay in tumor growth in a B16-F10 murine melanoma model [74].

Second, the mobilization of BM-derived CEPs may contribute to vasculogenesis. Some chemotherapy drugs can inhibit VDA-induced mobilization of BM-derived CEPs [35, 89]. Thus, combining these drugs with VDAs may amplify the antitumor effects of VDAs. For example, administration of cyclophosphamide (CTX) on a regular low-dose metronomic schedule resulted in a consistent decrease in both the level and viability of CEPs, and enhanced inhibition of tumor growth in human lymphoma-bearing mice [90]. When the VDA OXi4503 was combined with CTX to treat primary orthotopic tumors in mice in a metronomic manner, a reduction of the residual rim and enhanced inhibition of tumor growth was observed due to the suppression of the CEP spike and tumor colonization induced by OXi4503 [75]. Furthermore, low-dose metronomic chemotherapy could induce a transient normalization of functional tumor vessels [87] that may improve the delivery of chemotherapeutic agents. However, only certain chemotherapeutic agents such as CTX and gemcitabine can suppress the mobilization and increase in CEPs [35]. Other agents including paclitaxel, docetaxel, and 5-fluorouracil do not inhibit CEP mobilization but can induce a rapid increase in CEPs shortly after treatment [35].

Third, the synergistic effect generated by combining VDAs and chemotherapeutic agents can be affected by the schedule and sequence of administration of the two agents. Theoretically, chemotherapy should be administered before the VDA. This would allow the cytotoxic drug to be well distributed throughout the tumor through the vasculature, and then trapped once the blood vessels are shutdown by the VDA [9, 91]. Maximum effects were achieved when cisplatin was administered 1 to 3 hours before the administration of a VDA [92]. Complications can results if a VDA is administered before chemotherapy. For example, Siemann et al. [92] reported impaired treatment efficacy when cisplatin was administered 1 hour after CA4P or 2 hours after DMXAA in a rodent KHT sarcoma tumor model. This may be explained by the fact that cisplatin delivery to the tumor may be impeded shortly after VDA-induced vessel shutdown, and some tumor cells may survive under hypoxic conditions after VDA treatment [93-95]. However, an enhanced antitumor effect, and even complete tumor remission, was achieved when paclitaxel was administered 24 hours after ZD6126 [96]. This relatively long interval may provide additional time for the tumor to develop VDA-induced central necrosis. Cytotoxic drugs can then be used to target the proliferating cells in the residual viable rim [73, 97-99]. Thus, pretreatment with a VDA may amplify the effects of cytotoxic agents on proliferating cells in the tumor periphery [91].

### Vascular disrupting agents combined with radiotherapy

Only a few clinical trials have evaluated the combination of VDAs with radiotherapy [76, 77], though preclinical studies have demonstrated the effectiveness of combination treatment for solid tumors [67]. The resistance of solid tumors to VDAs is primarily the result of residual viable cells in the periphery. These viable cells are likely to be in a well-oxygenated environment and actively proliferating, which makes them more sensitive to radiotherapy [69, 79, 100-103]. In addition, tumor cells in the G2/M phase of the cell cycle are more sensitive to radiotherapy, and VDAs can induce tumor cell arrest in the G2/M phase [67].

Treatment with VDAs can induce central necrosis. Thus, regions of hypoxia and acidosis develop within the tumor. Because oxygen is critical for effective radiation-induced DNA damage, hypoxia and acidosis may impair the effects of radiotherapy on tumors [67]. Therefore, it is generally optimal to perform radiotherapy before administering a VDA [67]. The first report of such a combination by Li et al. [104] showed additive effects in a murine tumor model. An enhanced antitumor effect has been observed with a range of VDAs (DMXAA, CA4P and OXi4503) combined with single or fractionated irradiation in a C3H murine mammary carcinoma model [105]. Indeed, some studies have confirmed a reduced effect when VDAs were administered prior to irradiation [95]. However, there are some exceptions. For instance, OXi4503 or ZD6126 yielded an increased response when administered before radiotherapy [105, 106]. In KHT sarcomas, an improved antitumor effect was observed whether ZD6126 was administered before or after radiotherapy [92, 106, 107]. The discrepancy in therapeutic responses may be related to the different VDAs and different tumor models used in these studies [99].

Interestingly, combination therapy has been particularly effective for the treatment of large tumors, which are normally less sensitive to radiotherapy due to hypoxia and acidosis caused by increased interstitial pressure and impaired blood flow [67, 107, 108]. However, VDAs cause extensive central necrosis in tumors and allow elimination of residual rim cells with radiotherapy [9].

### Vascular disrupting agents combined with targeting molecules labeled with ^131^iodine

Two strategies for combining VDAs with targeted internal irradiation have been pursued, in light of tumor resistance to VDAs and the limited efficacy of radioimmunotherapy for the treatment of solid tumors (due to poor penetration into the central regions of large tumors) and the relative resistance of hypoxic tissue to radiotherapy) [67].

## VASCULAR DISRUPTING AGENTS IN COMBINATION WITH RADIOIMMUNOTHERAPY

The combination of VDAs and radioimmunotherapy has been evaluated using a carcinoembryonic antigen (CEA)-positive colorectal xenograft model [78]. The study showed that the combination of the ^131^I-A5B7 anti-CEA antibody with CA4P eliminated five of six tumors. In another animal experiment, the retention of the ^131^I-A5B7 anti-CEA antibody was extended for up to 4 days in an SW1222 colorectal xenograft when CA-4P was administered 2 days after the antibody [109]. Based on these preclinical studies, a phase I clinical trial was performed using the same combination strategy (i.e., ^131^I-A5B7 anti-CEA antibody and CA4P) in patients with gastrointestinal adenocarcinoma [77]. However, only one of 10 patients showed a partial response. Although tumor-specific uptake of ^131^I-A5B7 was demonstrated, no direct comparison of the absorbed doses between the target organs and the tumor was provided. In addition, dose-limiting myelosuppression was observed in heavily pretreated patients.

## VASCULAR DISRUPTING AGENTS IN COMBINATION WITH A NECROSIS-TARGETING MOLECULE LABELED WITH ^131^IODINE

Recently, a specific antitumor approach has been proposed that involves combining a natural necrosis-targeting molecule labeled with ^131^iodine with CA4P [82]. Hypericin, a polycyclic aromatic compound, is a naturally occurring chromophore extracted from the plant genus *Hypericum perforatum* commonly known as St. John's Wort [110]. Hypericin has been used extensively in photodynamic therapy as a potent photosensitizer due to its high photo-oxidative cellular damaging effect [111-113]. However, the applications are limited to only superficial tumors because the toxic functions must be activated by an external light source.

More recently, Ni and colleagues [114-118] discovered that hypericin has a strong necrosis affinity. Although the mechanism responsible for the necrosis-avid effect has not been fully elucidated, one possibility is that hypericin may bind to phosphatidylserine and phosphatidylethanolamine in the lipid bilayer of the cell [83, 119]. The necrosis-avid feature of hypericin is independent of its photosensitivity. In addition, a series of radiolabeled hypericin derivatives such as ^123^I-iodohypericine and ^131^I-iodohypericine have shown a similar necrosis affinity in several infarction and intratumoral necrosis animal models [114-117]. Therefore, there has been increasing interest in hypericin as a potential necrosis-targeting therapy. Van de Putte et al. [114] verified the necrosis avidity of hypericin and the radiotherapeutic effect of ^131^I-hypericin in nude mice bearing radiation-induced fibrosarcoma (RIF-1). Significant delays in tumor growth were observed in the fluorodeoxyglucose micro-positron emission tomography group compared to the control group.

Ni et al. [80-82] took advantage of the necrosis-avid feature of hypericin and designed a novel anticancer theranostic strategy that combined a VDA (CA4P) and ^131^I labeled hypericin (^131^I-Hyp). In this sequential dual-targeting approach, a VDA is used to disrupt the tumor vessels (the first target) in solid tumors and cause massive necrosis. Following VDA treatment, ^131^I-hypericin is injected intravenously and reaches the necrotic zone based on its strong necrosis avidity. Accumulated ^131^I-hypericin in CA4P-induced necrotic zones may kill residual cancer cells (the second target) with ionizing radiation and significantly inhibit tumor relapse. The necrotic avidity of ^131^I-hypericin was remarkable, with a necrotic target-to-liver ratio of more than 20 times, which was approximately 100 times the cumulative dose of 50 Gy that is necessary to elicit a tumor response to radiotherapy. More recently, ^131^I-hypericin was administered 24 hours after CA4P in a rabbit model of multifocal VX2 tumors [84]. The results demonstrated the high targetability of ^131^I-hypericin to tumor necrosis by in vivo single-photon emission computed tomography. The accumulation of ^131^I-hypericin was 98 times higher in necrotic tumor areas compared to viable tumors and other organs by gamma counting, and was confirmed by autoradiography and fluorescence microscopy. The necrosis-targeting effect persisted for more than 9 days. Tumor growth was significantly reduced and the doubling time was significantly increased in response to combined VDA ^131^I-hypericin treatment.

Given that necrosis is common in solid tumors treated with anticancer therapies, this sequential dual-targeting approach may be a novel solution to the problem of tumor resistance to VDA therapy. Another advantage of this strategy is that the residual viable tumor cells following VDA treatment may not only be eradicated, but also can be visualized with nuclear imaging modalities as radiolabeled hypericin, which exhibits superb sensitivity in targeting necrotic tissues [114].

## SUMMARY

In summary, preclinical studies and clinical trials have demonstrated the existence of a residual viable tumor rim after treatment of solid tumors with VDAs. Thus, tumor cells may survive despite vascular disruption and recur, which is suggestive of tumor resistance to these therapeutic agents. Although several mechanisms have been proposed to explain tumor resistance, none of them can explain the entire phenomenon. A variety of MRI markers, particularly functional parameters, can be obtained to visualize and quantify the process of tumor resistance. Many efforts have been made to improve the antitumor effects of VDAs. Current strategies to prevent tumor resistance mainly emphasize the combination of VDAs with other approaches including antiangiogenic agents, chemotherapy, radiotherapy, radioimmunotherapy, and sequential dual-targeting internal radiotherapy. Most of these combination therapies have demonstrated promising effects towards combating the residual viable tumor rim. However, much work remains before they can be incorporated into routine clinic practice.
